# Nonlinear EEG Complexity as a Marker of Maladaptive Brain Plasticity in Substance Use Disorders: A Multi-Group Machine Learning Classification Study

**DOI:** 10.3390/brainsci16060603

**Published:** 2026-05-31

**Authors:** Mashal Fatima, Faraz Akram, Imran Khan Niazi

**Affiliations:** 1 Department of Biomedical Engineering, Riphah International University, Islamabad 44035, Pakistan; mashal.fatima@riphah.edu.pk (M.F.); faraz.akram@riphah.edu.pk (F.A.); 2 Centre for Chiropractic Research, New Zealand College of Chiropractic, Auckland 1060, New Zealand; 3 Faculty of Health & Environmental Sciences, Health & Rehabilitation Research Institute, Auckland University of Technology, Auckland 1010, New Zealand; 4 Centre for Sensory-Motor Interactions, Department of Health Science and Technology, Aalborg University, 9220 Aalborg, Denmark

**Keywords:** nonlinear EEG complexity, substance use disorder, maladaptive brain plasticity, EEG, frontal cortex

## Abstract

**Highlights:**

**What are the main findings?**

- Across six substance use groups and control participants without a diagnosis of substance use disorder (*n* = 350; 50 per group), all four nonlinear EEG complexity measures (LLE, FD, HE, KC) were significantly reduced in users, with the largest losses in frontal and central regions linked to motor planning and sensorimotor integration.
- A K-Nearest Neighbour classifier separated the seven groups with 98.4% accuracy (100% sensitivity, 96.8% specificity), showing that different substance profiles produce distinct EEG signatures of maladaptive cortical plasticity.

**What are the implications of the main findings?**

- This is the first systematic multi-group evaluation of nonlinear EEG complexity across diverse substance use profiles within a unified classification framework, supporting the interpretation of complexity loss as an electrophysiological marker of maladaptive brain plasticity rather than a generic abnormality measure.
- Because EEG is portable, low-cost, and resistant to the manipulation that limits blood, urine, and self-report screening, the approach is well suited to baseline plasticity assessment at intake, longitudinal monitoring during neurorehabilitation, and substance-specific personalization of treatment.

**Abstract:**

Background: Chronic exposure to addictive substances induces persistent alterations in neural dynamics, reflecting maladaptive brain plasticity. While such changes are well documented using neuroimaging techniques, their electrophysiological signatures—particularly those derived from nonlinear EEG complexity—remain insufficiently explored across diverse substance use profiles. This preliminary study aims to investigate whether nonlinear EEG complexity measures can serve as sensitive biomarkers of maladaptive plasticity in substance use disorder (SUD) across multiple substance categories. Methods: A total of 350 participants were included and categorized into seven groups (*n* = 50 each): six substance use groups (cannabis, heroin, heroin–cannabis, methamphetamine–cannabis, methamphetamine–heroin, and multi-drug) and one control group without a diagnosis of substance use disorder. Resting state EEG signals were recorded using an eight-channel system. Four nonlinear features, Largest Lyapunov Exponent (LLE), Fractal Dimension (FD), Hurst Exponent (HE), and Kolmogorov Complexity (KC) were extracted. Statistical analysis was performed using two-way ANOVA, and classification was conducted using the K Nearest Neighbour (KNN) algorithm. Results: Significant group differences (*p* < 0.05) were observed across all nonlinear features. Control participants without a diagnosis of substance use disorder consistently exhibited higher complexity values compared to substance use groups, indicating reduced neural dynamical variability associated with the history of sustained substance uses over multiple years. Region wise analysis revealed that frontal and central cortical areas linked to motor planning and sensorimotor integration were particularly affected. The KNN classifier achieved an accuracy of 98.4%, sensitivity of 100%, and specificity of 96.8%. Conclusions: Nonlinear EEG complexity measures provide a robust electrophysiological marker of substance induced maladaptive brain plasticity. The observed reduction in complexity reflects impaired neural adaptability, particularly within motor control networks. These findings highlight the potential of EEG based complexity metrics for objective assessment, classification, and neurorehabilitation monitoring in substance use disorders.

## 1. Introduction

Globally, substance use has shown a substantial increase, with over 296 million individuals reporting drug consumption in 2021, representing a 23% rise compared to the previous decade [1]. Among these, approximately 219 million individuals use cannabis and 60 million use opioids, making them the most widely consumed substances worldwide. Substance Use Disorder remains a critical public health challenge, involving the chronic misuse of substances such as cannabis, heroin (opioids), and methamphetamine (Meth). Prolonged exposure to these substances induces persistent alterations in neural dynamics, reflecting maladaptive brain plasticity, which underlies cognitive, behavioral, and motor impairments. Understanding these neurophysiological changes is essential for developing objective diagnostic and rehabilitation strategies.

Electroencephalography (EEG) has emerged as a powerful and accessible modality for investigating the electrophysiological correlates of substance induced brain alterations [2,3,4,5,6,7,8,9,10]. Compared to neuroimaging techniques such as Magnetic Resonance Imaging, Positron Emission Tomography, and Magnetic Resonance Spectroscopy, EEG offers superior temporal resolution, portability, and cost effectiveness, making it particularly suitable for large scale screening and resource constrained settings. These advantages enable real time monitoring of brain activity and facilitate the study of dynamic neural processes associated with substance abuse.

Substance use disorders (SUD) arise from a complex interaction of anatomical [3,11], physiological [5,6,7,8,9,10], psychological [6], and environmental factors, making their comprehensive assessment challenging. Conventional detection approaches including blood, urine, saliva, and hair analysis—are often limited by inaccuracies and reliance on self-reporting. Consequently, there is growing interest in EEG based methods that assess substance induced brain dysfunction directly [4,5,6,7,8,9,10]. Prior EEG studies have primarily focused on three domains: (i) functional brain connectivity [6,7,8,9], (ii) neural responses to external stimuli [10], and (iii) identification of electrophysiological biomarkers [4,5,12,13,14,15,16]. These biomarkers have been utilized to develop EEG based classification framework, employing measures such as entropy [5,13], Lempel–Ziv complexity [12], power spectral indices [15], and recurrence quantification analysis [16], achieving high classification accuracies (e.g., 97% in heroin users vs. controls (no SUD diagnosis) [5]).

Despite recent advances in EEG-based assessment of substance use disorders, conventional linear EEG analysis techniques remain limited in describing the inherently complex and nonlinear behaviour of brain activity. Chronic substance exposure alters cortical neural dynamics in a manner that may not be fully characterized using linear correlations or spectral power analysis alone. In contrast, nonlinear EEG complexity measures provide a more comprehensive framework for evaluating the irregular, dynamic, and adaptive properties of neural signals [17,18]. These measures are particularly useful for identifying substance-related alterations in cortical organization because they capture variations in neural adaptability, chaoticity, signal complexity, and long-range temporal dependency. Consequently, nonlinear EEG analysis may provide more sensitive electrophysiological markers for distinguishing different substance use groups and assessing altered cortical dynamical behaviour associated with chronic drug exposure. To date, the role of nonlinear complexity metrics in characterizing substance induced brain alterations across multiple drug categories remains underexplored. In this preliminary study, we investigate four nonlinear features—Largest Lyapunov Exponent (LLE), Fractal Dimension (FD), Hurst Exponent (HE), and Kolmogorov Complexity (KC)—to quantify EEG complexity across diverse substance use groups. By analyzing resting state EEG from cannabis, heroin, polysubstance, and multi drug users, this work aims to identify robust electrophysiological markers of maladaptive brain plasticity and evaluate their effectiveness for multi group classification. The findings are expected to provide insights into disrupted neural dynamics, particularly within motor control and sensorimotor integration networks, and to support the development of objective tools for diagnosis and neurorehabilitation monitoring. To the best of our knowledge, this is the first preliminary study to systematically evaluate nonlinear EEG complexity across multiple substance use groups within a unified classification framework, with direct interpretation in terms of maladaptive brain plasticity and motor control networks.

## 2. Materials and Methods

**Figure 1:**
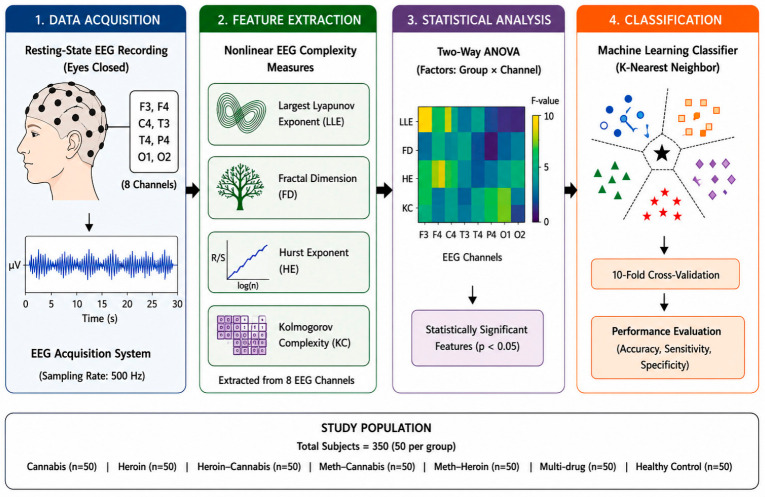
EEG based classification framework for nonlinear complexity analysis.

This study proposes an EEG based framework to characterize nonlinear brain dynamics associated with SUD. Resting state EEG signals were pre-processed, segmented, and analyzed using nonlinear feature extraction methods. Non-invasive EEG signals were acquired from control participants without a diagnosis of substance use disorder and substance use groups. In order to eliminate noise and artefacts from the signals, they were first filtered and pre-processed. Next, information about noteworthy characteristics was extracted using 2s segmented/epoch data. To convert EEG signals into useful data, several nonlinear feature extraction approaches were used. After that, a statistical test was used to order the characteristics based on their significance level. In order to determine the correctness of the suggested research, the ranking characteristics are finally fed into the classifier. The suggested technique is shown in Figure 1.

### 2.1. Study Protocol

This study in collaboration with the under mentioned rehabilitation centres was conducted at the various sites located in the vicinity of Islamabad and Rawalpindi, Pakistan namely:

1. Ali Rehab Center Islamabad ARC
2. Islamabad Rehab and Caring Centre.
3. Healing House Psychiatric Clinic, Drug Detoxification and Rehabilitation Centre.
4. Life Ways Rehab Centre.

Individuals with a history of SUD were selected as the case group, while age- and sex-matched volunteers without a diagnosis of substance use disorder served as the control group (CON). Before the study began, institutional ethics committee (Research Ethical Committee Riphah) approval was formally obtained. Before the patients and controls were included in the trial, their fully informed written consents were acquired.

*Exclusion criteria:* Individuals and controls who were also receiving antiseizure, rehabilitative, or methadone medications, had cooccurring epilepsy, or had other conditions that would alter the EEG and impede the effects of medication were not included. Individuals undergoing pharmacologic treatment for medication withdrawal. Patients failing to provide permission.

*Inclusion Criteria:* A neurologist attested to the individuals’ normal brain signals. No head injuries, trauma, comorbidity, or current DSM-5 axis based mental disorders in the past was recorded [19]. The physiologist also assessed possession of drug dependency criteria according to the DSM-5 report [19]. Participants without a diagnosis of substance use disorder who matched in terms of age and sex served as controls. The study included 350 subjects Demographics of the subjects recruited in the study is represented in Table 1.

### 2.2. Data Acquisition

To record EEG data, 8 Ag/AgCl electrodes were placed bilaterally on the subject’s scalp. The electrodes (F3, F4, C4, T3, and T4, P4, O1, and O2) were positioned in accordance with international standards 10–20 using an EEG acquisition equipment namely, OPEN BCI [20]. OpenBCI specializes in open-source brain computer interface technology, offering a range of products designed for neurotechnology research and development offering two types of boards with range of electrodes from 4 to 16 channels and 250 Hz sampling frequency.

EEG signals were collected, and their groups were classified according to the sort of substance addiction they had. Therefore 50 subjects in each group were recruited along with 50 controls. These were all males. Total of 3 min EEG signals were acquired for each subject with an alteration of 30 s eyes open and 30 s eye close paradigm. Last two minutes data was used for the pre-processing and feature extraction. All participants were assessed by rehabilitation professionals and clinicians prior to EEG acquisition to ensure the absence of active substance consumption at the time of data collection. In addition, baseline EEG recordings were obtained at a comparable stage of rehabilitation across participants, following the initial stabilization phase as recommended by the clinical staff at the rehabilitation facilities.

### 2.3. Pre-Processing

In EEG signal preprocessing for mining of meaningful data free of any motion artifact and noise is the most crucial step. Therefore, filtration was carried on the resting state EEG with a lead impedance artifact elimination using 50 Hz notch filter. Bandpass filter with a low pass cut-off sets at 40 Hz and high pass cut-off set at 0.5 Hz was applied. Eight channels of EEG signals were used for all the different groups of the subjects and control having a non-overlapping window size of 2 s.

### 2.4. Feature Extraction

Nonlinear dynamic analysis is valuable in capturing a meaningful information which can be used in different approaches [21,22] e.g., to detect seizures [23], detect heroin addiction [3], characterization of fibromyalgia [24], identification between normal and diabetic heart rate [25]. By analyzing the complex and chaotic properties of EEG signals, nonlinear methods can potentially detect early signs of substance impact, or subtle effects that linear methods might overlook. Four different nonlinear feature extractors, namely largest Lyapunov exponent (LLE) [26], fractal dimension (FD) [27], Kolmogorov complexity (KC) [28], and Hurst exponent (HE) [29] were implemented. Details of these feature extractors are given below.

**Largest Lyapunov exponent (LLE):** LLE is a nonlinear parameter used to evaluate the sensitivity of EEG signals to small changes in initial conditions and is commonly used to quantify chaotic neural behaviour [26]. Higher LLE values indicate greater signal divergence and reduced predictability, reflecting increased cortical complexity. Alterations in LLE may indicate disrupted neural adaptability associated with chronic substance exposure. Mathematically it can be formulated as:

(1) $$\lambda =\underset{t\to \infty }{\lim}\frac{1}{t}\;log\frac{∥\delta Y(t)∥}{∥\delta Y(0)∥}$$

In a healthy brain, a moderate level of chaos is considered beneficial because it reflects flexible and adaptive neural processing. However, chronic substance use can disrupt this balance by reducing the brain’s ability to adapt efficiently, leading to altered cortical dynamics and changes in LLE values. Therefore, LLE can serve as a useful biomarker for identifying disruptions in neural dynamical organization associated with substance dependence.

**Fractal dimension (FD):** FD serves as a quantitative metric for assessing the complexity of signals and unveiling the presence of homogeneous patterns within them. As FD values rise, the level of complexity in the signals also increases [27]. Higher FD values generally correspond to increased signal complexity and irregular neural activity, whereas lower FD values indicate reduced dynamical variability and diminished cortical adaptability. Previous studies have reported decreased fractal complexity in several neurological and psychiatric disorders, suggesting impaired neural communication and reduced dynamical efficiency. Mathematically:

For a time, series x(t), compute the length of the signal over different segment lengths m:

(2) $$L\left(m\right)=\frac{1}{k}\sum_{j=1}^{K}1/m\sum_{k=1}^{m}\left|x\left(k\right)-x\left(k+jm\right)\right|$$

Then computing the FD as:

(3) $$DH=\underset{m\to 0}{\lim}\frac{log(L\left(m\right))}{log(m)}$$

**Kolmogorov complexity (KC):** According to [28], KC calculates the signals’ complexity. Signals with higher KC values exhibit greater variability and less repetitive structure, indicating more complex brain dynamics. In contrast, lower KC values suggest more regular and predictable neural behaviour, which may reflect reduced cortical adaptability. Alterations in KC have been associated with impaired neural processing in several neurological and psychiatric conditions. Therefore, KC provides valuable insight into the disruption of neural complexity associated with chronic substance exposure. The values of the KC, k(x) for any length p, characteristics increase with signal randomness and can be computed as given in Equation (4)

(4) $$k\left(x\right)=\min\left\{|p|:U\left(p\right)=x\right\}$$

where, U is a universal Turing machine that runs the algorithm and gives an output coefficient x.

**Hurst exponent (HE):** According to reference [29], HE is a statistical measure used to forecast when information will recur in EEG signals. It utilizes the non-overlapping segment’s (T) mean, standard deviation and range (R) to find the HE. Equation 5 describes it mathematically as

(5) $$H\approx \underset{T\to \infty }{\lim}\frac{log(R(T))}{log(T)}$$

In EEG analysis, HE is useful for assessing the complexity and temporal organization of brain dynamics. Higher HE values indicate persistent behaviour, where future signal trends are likely to continue in the same direction, whereas lower values suggest anti-persistent or more random behaviour. Healthy neural systems generally exhibit balanced temporal correlations that support stable and adaptive brain functioning.

The nonlinear EEG features used in this study were selected to characterize different aspects of cortical dynamics associated with substance use disorders. The selected methods were used for feature extraction because they represent complementary properties of neural activity, including chaoticity, geometrical complexity, signal irregularity, and long-range temporal dependency. Resting-state EEG signals were segmented into non-overlapping 2-s epochs to ensure stable temporal analysis.

Since 2 min (120 s) of EEG data were analysed for each subject, the total number of epochs generated per subject was:

Epochs per subject = 120 s/2 s = 60

For each epoch, four nonlinear features were extracted from all eight EEG channels. Therefore, the total number of extracted features per epoch was:

Features per epoch = 8 channels × 4 features = 32

Consequently, the total number of feature instances generated for each subject was:

Feature instances per subject = 60 epochs × 32 features = 1920

Considering the complete dataset of 350 participants, the overall nonlinear EEG feature space analysed in this study was:

Total feature instances = 350 × 60 × 32 = 672,000

Thus, the proposed framework analysed a large-scale nonlinear EEG dataset consisting of 672,000 feature values derived from multichannel resting-state EEG recordings, enabling robust statistical analysis and multi-group classification of substance use disorders.

### 2.5. Statistical Analysis

Statistical analysis was performed to evaluate the significance of the extracted nonlinear EEG features among the six substances use groups and control (no SUD diagnosis). Since nonlinear features were extracted from multiple EEG channels and subject groups, a two-way ANOVA was applied to analyse variations in feature values with respect to both group category and channel-wise distribution. The analysis was conducted on the four extracted nonlinear features.

The primary objective of the statistical analysis was to determine whether chronic substance exposure produced significant alterations in EEG complexity measures across cortical regions. A significance threshold of *p* < 0.05 was used to identify statistically relevant differences between control (no SUD diagnosis) and substance use groups. Features demonstrating significant differences were subsequently utilized for classification analysis. The statistical findings confirmed that nonlinear EEG complexity measures varied significantly across the study groups, supporting their suitability as discriminative biomarkers for substance use disorder classification and assessment of maladaptive brain plasticity.

### 2.6. Classification

EEG data classification is done with supervised, unsupervised, probabilistic, and deep learning approaches [30,31,32]. Artificial Neural Networks (ANN) [24], Support Vector Machine (SVM) [23], Decision Tree (DT) [33], K Nearest Neighbor (KNN) [34], Naïve Bayes (NB) [35], and Convolutional Neural Network (CNN) [4] algorithms were used to address classification problems. Among these few classifiers depends on hardware and cannot work with small data. Commonly used classifier like DT creates expectations based on outcomes that may cause inaccuracy. In KNN, the computational cost is high but can classify big data. NB provides less accurate results. CNN requires a large dataset for analysis and has a high computational cost [4]. SVM is a standard supervised classification method that is not new but is still a powerful tool for classification well in many cases like biomedical settings due to their tendency not to over fit. In this study ensemble, KNN and SVM classifier were used.

SVM, KNN, and ensemble subspace classifiers share the ability to handle high dimensional EEG data effectively. They focus on accurate classification of brain activity patterns and can adapt through feature selection. All three methods emphasize generalization, making them versatile for various EEG analysis tasks like seizure detection and cognitive state classification.

The classification framework used in this study consisted of seven classes, including six substances use groups and control (no SUD diagnosis). The extracted nonlinear EEG features obtained from eight EEG channels were analyzed using the three classifiers to distinguish between six substances use groups and control (no SUD diagnosis). Only statistically significant features identified through two-way ANOVA (*p* < 0.05) were used for classification analysis. KNN achieved the highest performance by effectively identifying local similarity patterns within the nonlinear EEG feature space, indicating well-defined class-specific feature distributions among the study groups. Linear SVM analyzed the global separation boundaries between classes within the multidimensional feature space; however, some overlap among feature distributions of different substance groups reduced linear separability. The Ensemble Subspace Discriminant classifier further analyzed the data using multiple discriminant learners trained on randomized feature subsets, which improved classification robustness and reduced overfitting.

A 10-fold cross validation strategy was used in this study, which is a common method in machine learning and data analysis for evaluating a classification model’s effectiveness [36]. The dataset is split into ten equal sized subgroups, or “folds,” for the 10-fold cross validation. Nine folds are used to train the classification model in each iteration of the cross-validation procedure, and the final fold is saved as a validation set for evaluating the model’s performance. Ten times this process is performed, with one of each fold acting as the validation set. For each fold, the performance measures including accuracy, precision, recall, and F1 score were computed. The performance of the classification model was then assessed overall using the average performance across all folds. For KNN classification, the number of nearest neighbors was set to k = 5 using Euclidean distance as the similarity metric. The Linear SVM classifier was implemented using a linear kernel with standardized feature normalization. For the Ensemble Subspace Discriminant classifier, multiple discriminant learners were trained on randomized subsets of the nonlinear EEG feature space to improve classification robustness and reduce overfitting during multiclass analysis.

## 3. Results

**Figure 2:**
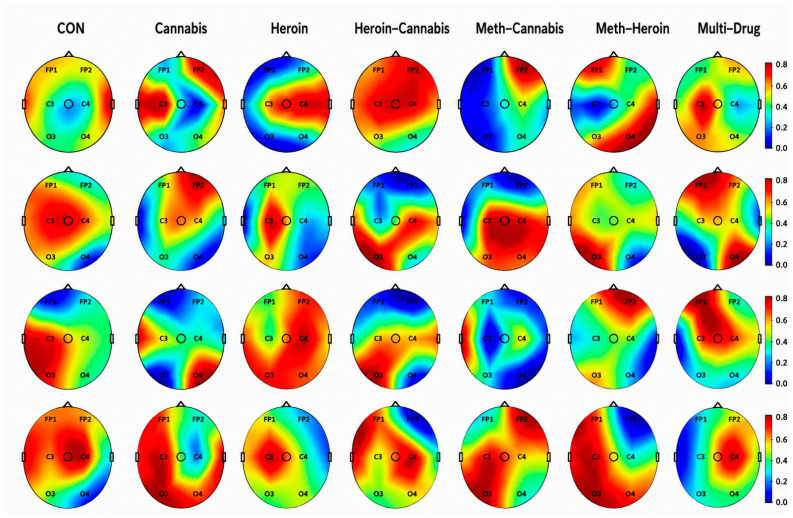
Topographic plots for the nonlinear complexity parameters for different categories of addicts and control (no SUD diagnosis). First row is the feature plot of LLE, then FD, HE and KC is the last row. CON = control (no SUD diagnosis), LLE = Largest Lyapunov Exponent; FD = Fractal Dimension; HE = Hurst Exponent; KC = Kolmogorov Complexity.

Significant statistical differences and feature based variations in brain activity patterns were observed between substance use groups and the control group, as illustrated in Figure 2. The Cannabis group, which may be impacted by cannabis use, shows increased electrical activity, complexity, and asymmetric distributions in particular areas. On the other hand, the brain activity of the heroin group shows a variety of predictable and complicated patterns. The heroin-cannabis combination has diverse impacts on various brain areas, as seen in the Heroin Cannabis group. While the Meth-Heroin group shows fluctuations in complexity, the Meth-Cannabis group has more consistent brain activity patterns. The multi-drugs category emphasizes the variety and complexity of effects on several brain regions. These results highlight the complex and diverse effects that different drugs have on brain activity, emphasizing the significance of comprehending these patterns for both medical and public health reasons.

In general, signals from healthy biological systems tend to have greater complexity characteristics, while states of addiction are associated with lower complexity. Topographic distribution of nonlinear EEG complexity measures for Largest Lyapunov Exponent (LLE), Fractal Dimension (FD), Hurst Exponent (HE), and Kolmogorov Complexity (KC) across all substance use groups and control group (no SUD diagnosis) can be seen in Figure 2. The first row represents LLE, followed by FD, HE, and KC in subsequent rows. The topographic plots clearly demonstrate a consistent reduction in EEG complexity across substance use groups compared to controls (no SUD diagnosis), as evidenced by lower intensity distributions across multiple cortical regions. In contrast, control group (no SUD diagnosis) exhibit higher and more spatially distributed complexity patterns, indicating greater neural dynamical variability. Notably, frontal and central regions show pronounced reductions in complexity, supporting the study’s central claim that chronic substance exposure leads to maladaptive brain plasticity affecting motor control and sensorimotor integration networks. These spatial patterns further corroborate the statistical findings and reinforce the role of nonlinear EEG features as sensitive biomarkers of substance induced neural dysfunction.

**Figure 3:**
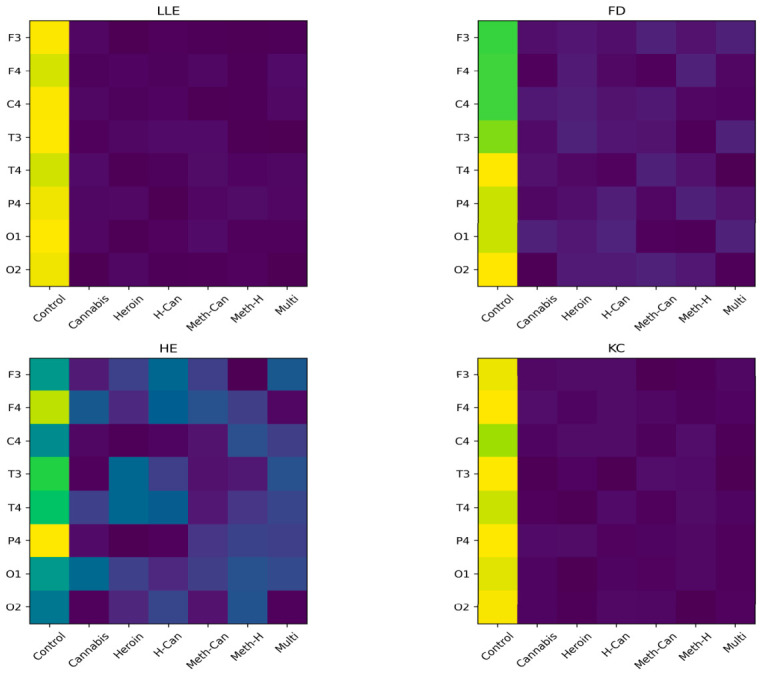
Channel wise visualization of nonlinear EEG complexity measures—Largest Lyapunov Exponent (LLE), Fractal Dimension (FD), Hurst Exponent (HE), and Kolmogorov Complexity (KC)—across seven study groups.

Channel wise visualization can be seen from Figure 3 of nonlinear EEG complexity measures—Largest Lyapunov Exponent (LLE), Fractal Dimension (FD), Hurst Exponent (HE), and Kolmogorov Complexity (KC) across seven study groups. Each subplot represents the spatial distribution of feature values across eight EEG channels (F3–O2). The observed nonlinear EEG complexity variations demonstrated distinct substance-specific patterns across the study groups. Controls (no SUD diagnosis) generally exhibited relatively stable and higher nonlinear complexity values, reflecting greater neural adaptability and organized cortical dynamics. In contrast, substance use groups demonstrated altered nonlinear behaviour characterized by variations in signal irregularity, chaoticity, and temporal dependency. Among the extracted features, FD and KC showed comparatively larger variations among different substance groups, indicating their stronger discriminative capability for multiclass classification. These reductions are particularly prominent in frontal (F3, F4) and central (C4) regions, supporting the central hypothesis that chronic substance exposure leads to maladaptive brain plasticity affecting motor control and sensorimotor integration networks. The figure complements topographic plots and statistical findings, providing a comprehensive spatial and group level representation of altered brain dynamics.

**Figure 4:**
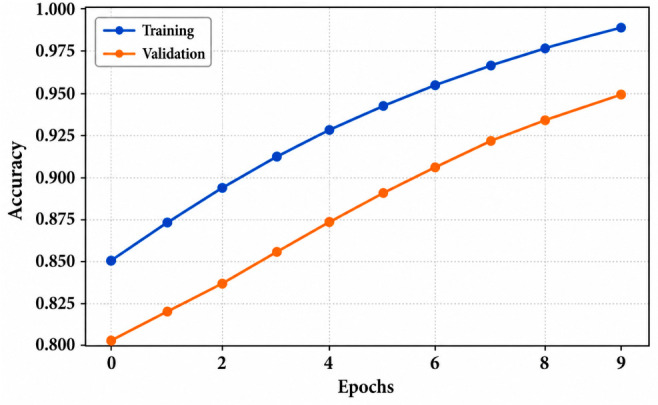
The learning curve shows convergence between training and validation performance, indicating that the model generalizes well and does not suffer from significant overfitting.

The learning curve shows convergence between training and validation performance, indicating that the model generalizes well and does not suffer from significant overfitting. As seen in Figure 4. This is particularly important given the high classification accuracy achieved. For further analysis, channel averaged comparison figures for the four nonlinear EEG features, namely Largest Lyapunov Exponent (LLE), Fractal Dimension (FD), Hurst Exponent (HE), and Kolmogorov Complexity (KC) are shown below. Across all four measures, the control group generally exhibited higher mean feature values than the drug dependent groups, indicating greater signal complexity and more preserved neural dynamics in non-addicted subjects. This overall pattern supports the premise that substance dependence is associated with a reduction in EEG complexity.

The analysis of nonlinear EEG features revealed significant differences between controls and various substance use groups. Table 2 presents the mean and standard deviation values of four nonlinear features—LLE, KC, FD, and HE —across 8 EEG channels for all groups. The extracted nonlinear EEG feature vectors preserved distinguishable distributions among the study groups, enabling effective multiclass classification using machine learning approaches. The classification analysis was performed to evaluate how efficiently nonlinear EEG complexity measures could differentiate substance-specific cortical dynamical patterns.

A clear trend was observed where the control group consistently exhibited higher values of LLE, FD, HE, and KC across most channels. These elevated values suggest greater signal complexity and variability in healthy individuals, indicative of normal brain dynamics. In contrast, reduced feature values in the drug dependent groups reflect diminished neural complexity, a hallmark of brain function deterioration due to substance abuse. Notably, cannabis users showed localized increases in complexity, especially in frontal regions, potentially due to altered functional connectivity patterns.

Among the addict subgroups, the multi-drug users showed the most reduced and inconsistent feature values, underscoring the extensive neural disruption caused by polysubstance abuse. Heroin and methamphetamine users displayed lower complexity particularly in central and occipital regions, aligning with literature on gray matter reduction and impaired connectivity in these areas.

Table 3 summarizes the classification performance of three machine learning models. The K Nearest Neighbor (KNN) classifier achieved the highest accuracy of 98.4%, with perfect sensitivity (100%) and high specificity (96.8%), yielding an F1 score of 98.48%. The Ensemble Subspace Discriminant model followed closely with 98.1% accuracy, while the Linear Support Vector Machine (SVM) yielded 96.9% accuracy. These results confirm the robustness of nonlinear EEG features in distinguishing substance use groups from controls and among different substance abuse categories.

## 4. Discussion

### 4.1. Nonlinear EEG Complexity and Substance Classification

The central finding of this study is that chronic substance use is associated with systematic reductions in nonlinear EEG complexity, and that these reductions are most pronounced in cortical regions involved in motor planning and sensorimotor integration. We interpret these complexity reductions as electrophysiological signatures of maladaptive brain plasticity, where chronic drug exposure drives neural systems toward less flexible, less adaptive operating regimes. This study is distinctive in covering six substance use categories alongside controls (Table 4), whereas previous studies have typically examined only one or two substance types (Table 4). The high classification accuracy (98.4% with KNN) demonstrates that different substances produce distinct patterns of maladaptive reorganization detectable at the scalp level. These nonlinear complexity features (LLE, FD, HE, KC) were extracted from 350 subjects across 7 groups (6 subjects and 1 control (no SUD diagnosis) group), statistically evaluated, and classified. The ensemble subspace discriminant and KNN classifiers achieved the highest accuracy of 98%, outperforming prior studies on smaller and less diverse cohorts. Furthermore, the classification analysis confirmed that nonlinear EEG complexity measures preserved sufficient discriminative information for effective multiclass differentiation among the study groups.

The typical operation of the brain demands a specific degree of organization and dynamic equilibrium of neural activity. Measurements of brain complexity are employed to assess these dynamic variations in brain activity. An increase in the complexity of electrophysiological activity indicates that different brain regions are not functioning in harmony, resulting in heightened noise in human brain activity. This augmented level of unpredictability in neural signals, which is observable in cannabis users, may be a consequence of reduced resting state functional connectivity, a phenomenon that has been observed in individuals with chronic cannabis use [37]. It is noteworthy that various studies have identified an increasing trend in can users’ complexity, which can be inferred from Figure 2 that FD values for the can user in all groups, H-can, meth-can and MD have increased complexity at the frontal lobes as compared to control and other groups.

### 4.2. Comparison with Previous Studies

Lapervote V et al. [12] demonstrated increased brain complexity in acute Can users, this trend can be observed in can users group for all the complexity parameters. These findings suggest that nonlinear EEG measures are sensitive to substance-specific alterations in cortical neural dynamics and may effectively differentiate cannabis-related neural activity patterns from other substance categories.

The basal ganglia also play a role in cognitive functions like decision making, planning, and impulsivity. Changes in gray matter volume might influence cognitive processes in drug addiction as seen in results of can abusers. The increased values in the frontal central and temporal regions correlate to the findings [38]. However, because the present study utilized an eight-channel EEG acquisition system, these regional observations should be interpreted cautiously as scalp-level electrophysiological variations rather than precise localization of underlying brain structures.

Yun K et al. [13] demonstrated a reduced EEG power spectrum in drug abusers’ brain complexity in Meth abusers that is consistent with a decrease in the FD and KC values in the Meth-Can with decrease at frontal, central and occipital regions.

KC values in heroin users were higher than in the CON group which correlate with the findings of a separate investigation where EEG signals from 75 individuals addicted to heroin were analyzed, and the entropy index was introduced as a diagnostic biomarker for heroin abusers [5]. Whereas the lower complexities at frontal, temporal, and central cortices in heroin addicted is due to the reduction of gray matter volume as suggested by [39].

In one study, EEG signal analysis was conducted on 39 cannabis users, with a comparison to 26 individuals in the control group. Increased signal complexity was found in 24 out of the cannabis users [12]. In most regions of the cerebral cortex, the FD, HE, and KC values of methamphetamine addicts were considerably lower than those of controls, indicating a reduction in the cerebral cortex’s complexity [40].

Reduced brain volume was observed in the hippocampus, Para hippocampus, frontal lobe, and temporal lobe when the brain structural differences between methamphetamine abusers and control group were compared [11]. This finding is consistent with a decrease in the complexity values at these specific sites for methamphetamine using groups. Decreased complexity in MD users shows that these people have difficulty concentrating and problem solving [41].

### 4.3. Classification Performance Analysis

The classification analysis demonstrated that nonlinear EEG complexity measures preserved distinct cortical dynamical patterns across different substance use groups. Control group (no SUD diagnosis) generally exhibited higher nonlinear complexity values, indicating greater neural variability and adaptive cortical organization, whereas substance use groups showed reduced complexity and altered neural dynamics. Among the extracted features, LLE reflected changes in chaotic neural behaviour, FD represented alterations in geometrical signal complexity, KC quantified reduced signal randomness, and HE characterized disrupted long-range temporal dependency. The observed variations in nonlinear EEG complexity suggest that chronic substance exposure produces substance-specific alterations in cortical dynamical organization, enabling effective multiclass discrimination among the study groups.

The superior performance achieved using KNN indicates that nonlinear EEG feature vectors formed compact and separable distributions within the multidimensional feature space, whereas the Ensemble Subspace Discriminant classifier demonstrated robust performance by preserving discriminative information across randomized feature subsets while reducing overfitting.

### 4.4. Clinical Implications

The nonlinear EEG complexity features identified here offer several advantages over traditional substance screening methods (blood, urine, self-report), including resistance to manipulation and the capacity to characterize neural state rather than merely confirming substance presence. Reduced EEG complexity reflects diminished neural adaptability and loss of dynamic flexibility, which are key neurophysiological signatures of maladaptive brain plasticity. These alterations suggest impaired reorganization capacity within cortical networks following chronic substance exposure, with particular relevance to motor control and sensorimotor integration circuits.

These findings support the potential of nonlinear EEG complexity as an objective biomarker for (a) initial assessment of plasticity state in patients entering rehabilitation, (b) longitudinal monitoring of plasticity recovery during treatment, and (c) tailoring rehabilitation strategies to the specific neural profile associated with each substance type. Future work should integrate nonlinear EEG complexity with direct motor performance measures (grip force, reaction time, gait analysis) and structural neuroimaging to build a multimodal picture of substance induced maladaptive plasticity and its recovery trajectory.

### 4.5. Limitations

Despite the promising classification performance achieved in this study, several limitations should be considered while interpreting the findings. The sample size, although sufficient to demonstrate meaningful classification capability, was relatively modest, with 50 participants in each group, and therefore larger cohorts may further strengthen the statistical robustness and generalizability of the results. In addition, all participants were male, which reduced biological variability within the dataset and enabled more consistent analysis; however, future studies including female participants are necessary to evaluate sex-related neurophysiological differences associated with substance use disorder. The study also focused on a limited number of substance categories, and some overlap existed among the polysubstance groups, reflecting the clinically realistic complexity of addiction patterns but potentially influencing the distinctiveness of EEG-based nonlinear feature distributions across classes. Furthermore, variability in the duration of drug abuse, participant demographics, and treatment or withdrawal stage during EEG acquisition may have contributed to inter-subject differences in nonlinear EEG complexity measures. Relatively broad distributions of nonlinear feature values observed within certain substance use groups may similarly reflect differences in addiction severity, duration of exposure, and individual neural adaptability. Although the eight-channel EEG system provided sufficient information to achieve effective nonlinear complexity analysis and classification, higher-density EEG recordings may further enhance the spatial characterization of cortical dynamics. Nevertheless, the proposed framework demonstrated reliable discrimination across multiple substance use groups using a relatively low-channel and clinically practical EEG setup, highlighting its potential applicability in objective neurophysiological assessment of substance use disorder. Future studies involving larger and more diverse multi-centre datasets, broader substance categories, longitudinal recordings, and multimodal neurophysiological assessments may further strengthen the clinical applicability and translational value of the proposed approach.

## 5. Conclusions

This study demonstrates that nonlinear EEG complexity measures provide a robust and physiologically meaningful framework for characterizing maladaptive brain plasticity associated with substance use disorder. By analyzing a large, multi-group cohort encompassing diverse substance use profiles, the findings reveal a consistent reduction in neural dynamical complexity, quantified through nonlinear complexity features (LLE, FD, HE, KC) in substance use groups compared to control group. These results indicate diminished neural variability and reduced adaptive capacity of cortical dynamics following chronic substance exposure.

Statistical analysis confirmed significant differences across all groups (*p* < 0.05), while regional patterns highlighted pronounced alterations in frontal and central brain regions, emphasizing the involvement of motor control and sensorimotor integration networks. These observations support the interpretation that substance-induced disruptions extend beyond cognitive domains and critically affect neural systems underlying coordinated motor function.

The high classification performance achieved, particularly by the K-Nearest Neighbour (KNN) classifier, demonstrates the strong discriminative capability of nonlinear EEG complexity features for distinguishing multiple substance use groups from controls. The findings indicate that nonlinear EEG measures preserve significant information related to altered cortical neural dynamics associated with chronic substance exposure. The proposed machine learning framework successfully differentiated substance-specific EEG patterns from resting-state recordings while cautiously presenting cortical alterations at the scalp level. Overall, the study highlights the potential of nonlinear EEG complexity analysis as a non-invasive and objective framework for substance use assessment, rehabilitation monitoring, and future AI-assisted neurophysiological evaluation.

From a translational perspective, the proposed approach offers a non-invasive, cost-effective, and scalable solution for substance use assessment. It holds promise for integration into clinical workflows, enabling improved diagnostic accuracy, monitoring of neurorehabilitation progress, and personalized intervention strategies.

Future work should explore longitudinal analyses, larger multi-centre datasets, and the integration of multimodal neuroimaging to further validate and extend these findings.

## Figures and Tables

**Table 1:** Demographics of the subjects recruited for the study.

Subjects (*n* = 50)	Age (Years) (M ± SD)	Lifetime Drug Use (Years) (M ± SD)	BMI (kg/m^2^) (M ± SD)
Con (Males)	30 ± 8	-----	24.8 ± 3.9
Cannabis (Males)	26 ± 4.2	5.5 ± 4.6	23.9 ± 4.1
Heroin (Males)	32.3 ± 9.2	6.5 ± 2.8	24.7 ± 4.5
Heroin-Cannabis (Males)	30.5 ± 8.7	5.5 ± 1.3	25.6 ± 4.2
Meth-Cannabis (Males)	32.3 ± 7.1	4.5 ± 2.3	26.3 ± 4.7
Meth-Heroin (Males)	30.5 ± 7.9	9.6 ± 7.6	27.2 ± 5.1
Multi-drugs (Males)	31.5 ± 12.6	8.2 ± 6	25.9 ± 5.0

Abbreviations: Con = control (no SUD diagnosis), SD = Standard Deviation; BMI = Body Mass Index.

**Table 2:** Mean ± SD of four features across eight EEG channels for each substance use group and the control (no SUD diagnosis) group.

Ch#_Feature	Control		Cannabis		Heroin		Heroin Cannabis		Meth Cannabis		Meth Heroin		Multi Drugs	
	Mean	SD	Mean	SD	Mean	SD	Mean	SD	Mean	SD	Mean	SD	Mean	SD
Ch7_LLE	0.3230	0.0055	0.0626	0.0199	0.1223	0.0322	0.0615	0.0984	0.0200	0.0138	0.1085	0.0562	0.2496	0.0469
Ch6_LLE	0.5953	0.0125	0.0684	0.0315	0.0115	0.0098	0.1264	0.1501	0.0600	0.0648	0.0116	0.0776	0.0211	0.0620
Ch4_LLE	0.2450	0.0182	0.0236	0.0187	0.0466	0.0304	0.1147	0.0625	0.0081	0.3553	0.2251	0.0982	0.0483	0.2428
Ch1_LLE	0.3233	0.0223	0.1866	0.1654	0.0530	0.0099	0.2885	0.0853	0.0924	0.0588	0.0995	0.0029	0.0560	0.3245
Ch5_LLE	0.3303	0.0812	0.1027	0.2157	0.0559	0.0225	0.0599	0.0216	0.0691	0.1382	0.0688	0.0231	0.0128	0.1427
Ch8_LLE	0.4459	0.0117	0.0462	0.0108	0.0379	0.0114	0.0302	0.0165	0.0304	0.1441	0.0424	0.0149	0.0339	0.0286
Ch3_LLE	0.0957	0.0047	0.1355	0.0214	0.0059	0.0011	0.0848	0.0177	0.0249	0.0125	0.0831	0.0033	0.0632	0.1556
Ch2_LLE	0.1990	0.0012	0.1778	0.0226	0.0320	0.0022	0.2063	0.0813	0.0679	0.0101	0.0177	0.0166	0.0104	0.1410
Ch5_HE	0.7788	0.0519	0.7301	0.0100	0.7213	0.0077	0.7159	0.0076	0.7381	0.1186	0.7471	0.0092	0.7190	0.0112
Ch6_HE	0.7889	0.0483	0.7323	0.0060	0.7210	0.0072	0.7105	0.0072	0.7349	0.1042	0.7519	0.0061	0.7173	0.0112
Ch1_HE	0.7616	0.0516	0.7274	0.0032	0.7178	0.0005	0.7059	0.0087	0.7365	0.1029	0.7368	0.0096	0.7302	0.0096
Ch7_HE	0.7684	0.0504	0.7250	0.0141	0.7200	0.0093	0.7062	0.0075	0.7279	0.0609	0.7376	0.0056	0.7104	0.0162
Ch8_HE	0.7794	0.0443	0.7503	0.0032	0.7208	0.0073	0.7101	0.0081	0.7371	0.0733	0.7441	0.0086	0.7175	0.0113
Ch4_HE	0.7836	0.0441	0.7283	0.0042	0.7217	0.0056	0.7128	0.0042	0.7285	0.0812	0.7440	0.0022	0.7195	0.0106
Ch2_HE	0.7728	0.0537	0.7314	0.0075	0.7162	0.0033	0.6955	0.0066	0.7392	0.0594	0.7388	0.0093	0.7117	0.0094
Ch3_HE	0.7656	0.0525	0.7285	0.0034	0.7198	0.0044	0.7218	0.0051	0.7334	0.0692	0.7500	0.0034	0.7198	0.0102
Ch6_KC	0.2500	0.0014	0.0567	0.0509	0.0856	0.0463	0.0677	0.0142	0.0186	0.0051	0.1071	0.0980	0.1547	0.2658
Ch8_KC	0.3124	0.0151	0.0371	0.0719	0.0574	0.0356	0.0915	0.0192	0.0622	0.0223	0.1121	0.0950	0.1492	0.2638
Ch1_KC	0.7114	0.0023	0.0820	0.0569	0.0818	0.0119	0.0955	0.0137	0.0075	0.0032	0.0500	0.0075	2.2208	0.1182
Ch3_KC	0.6801	0.0015	0.0537	0.0411	0.1374	0.0987	0.1541	0.1325	0.0205	0.0068	0.0313	0.0278	0.2023	0.0991
Ch4_KC	0.1908	0.0178	0.0962	0.0491	0.0227	0.0099	0.1070	0.0115	0.0295	0.0045	0.1128	0.0551	0.1433	0.2188
Ch2_KC	0.2862	0.0065	0.0030	0.0062	0.1349	0.0118	0.0234	0.0356	0.0696	0.0322	0.0336	0.0236	0.1972	0.1758
Ch7_KC	0.4631	0.012	0.0938	0.0188	0.0689	0.0125	0.0715	0.0207	0.0623	0.0598	0.0727	0.0574	0.2172	0.0127
Ch5_KC	0.4264	0.0014	0.0969	0.0119	0.0305	0.0152	0.1100	0.0311	0.1229	0.0499	0.0872	0.0506	0.1297	0.1966
Ch7_FD	0.0072	0.0114	0.0806	0.0079	0.0691	0.0091	0.0177	0.0024	0.0642	0.0146	0.0317	0.0102	0.0936	0.0144
Ch5_FD	0.5458	0.0960	0.0886	0.0064	0.0619	0.0043	0.0260	0.0028	0.0663	0.0102	0.0110	0.0098	0.0492	0.0061
Ch6_FD	0.5577	0.0976	0.0510	0.0059	0.0835	0.0042	0.0346	0.0059	0.0487	0.0112	0.0770	0.0061	0.0880	0.0061
Ch1_FD	0.4645	0.1995	0.0506	0.0053	0.0408	0.0030	0.0571	0.0059	0.0308	0.0151	0.0379	0.0010	0.0809	0.0050
Ch2_FD	0.2449	0.1995	0.0349	0.0037	0.0265	0.0010	0.0013	0.0023	0.0913	0.0111	0.0221	0.0098	0.0840	0.0063
Ch3_FD	0.3240	0.0901	0.0680	0.0003	0.0564	0.0041	0.0508	0.0054	0.0733	0.0117	0.0105	0.0120	0.0881	0.0050
Ch8_FD	0.1950	0.1528	0.0567	0.0046	0.0805	0.0006	0.0342	0.0032	0.0667	0.0105	0.0907	0.0100	0.0773	0.0051
Ch4_FD	0.2290	0.0758	0.2689	0.0032	0.2725	0.0010	0.1528	0.0435	0.1388	0.0108	0.2058	0.0099	0.0997	0.0067

LLE = Largest Lyapunov Exponent; FD = Fractal Dimension; HE = Hurst Exponent; KC = Kolmogorov Complexity; SD = Standard Deviation.

**Table 3:** The evaluation criteria for the classification of participants with SUD including control.

Classifier	Accuracy	Sensitivity	Specificity	Precision	F1 Score
KNN	98.4%	100%	96.8%	96.9%	98.48%
Ensemble Subspace Discriminant	98.1%	96.88%	100	100%	98.43%
Linear SVM	96.9%	93.75%	96.8%	96.7%	95.24%

KNN = K-Nearest Neighbor; SVM = Support Vector Machine.

**Table 4:** Comparison of studies on EEG based classification frameworks.

Study	Publication Year	Abuser Sample Size	Type of Substance Consumed	Extractive Index	Classifier	Accuracy (%)
[6]	2019	36	Meth	Weighted phase delay index, in the beta band	Support Vector Machine (SVM)	93
[9]	2013	36	Meth	Small global network characteristics	Improved probabilistic neural network	82.8
[8]	2015	49	Opioid (17), methadone treatment subjects (32)	Neucube model based on spiking neural network	Spiking neural network	86
[7]	2016	49	Opioid (17), methadone treatment subjects (32)	Neucube model based on spiking neural network	Spiking neural network	90
[12]	2017	39	25 Can users, 14 Can abusers	Lempel-Ziv Complexity	non-parametric Kruskall-Wallis ANOVA	↑ LZC in Can users
[13]	2012	48	Meth	Entropy	*t*-test	----- ↓ complexity
[14]	2020	57	Meth	microstates	linear regression model	temporal changes in microstate
[5]	2020	75	Heroin	Entropy	Perceptron neural network	97
[15]	2021	58	Opioid (20), Meth (15), people with alcohol use disorder (23)	Power spectral	Statistical analysis	--------
[4]	2021	45	Meth (mild: 15; moderate: 15; and severe: 15)	Convolutional Neural Network	Convolutional Neural Network and normalization	63.15
[10]	2014	23	cocaine	ERP components	Support Vector Machine (SVM)	75
[16]	2023	22	MD & CON	Recurrence Quantification Analysis	Support Vector Machine (SVM)	90
	**2026**	**350**	**6 substances use group & CON**	**LLE, FD, HE, KC**	**Ensemble subspace** **KNN**	**98.43** **98.48**

Meth = Methamphetamine; Can = Cannabis; CON = controls (no SUD diagnosis); MD = multi-drug users.

## Data Availability

Reasonable requests for data can be requested from the corresponding author but we will need to seek ethics committee approval prior to sharing any data.
